# Nitrogen plasma engineered MoS_2_ for catalyzing hydrogen evolution reaction

**DOI:** 10.1016/j.isci.2026.114714

**Published:** 2026-01-16

**Authors:** Haoyang He, Ai Wang, Fengrui Yang, Rui Shu, Xinyan Xie, Yongheng Wen, Dong Zhao, Mao Wang, Yijia Huang, Zhengwei Xie, Ling Li, Jianqi Zhu

**Affiliations:** 1 Key Laboratory of Micro-Nano Optoelectronic Materials and Devices at Sichuan Normal University of Sichuan Province, Chengdu 610101, China; 2 Optical Fiber Security Research Center, College of Smart Materials and Future Energy, Fudan University, Shanghai 200433, China; 3 College of Physics and Electronic Engineering, Sichuan Normal University, Chengdu 610101, China

**Keywords:** Electrochemistry, Applied sciences

## Abstract

Monolayer molybdenum disulfide (MoS_2_) possesses exceptional electrical characteristics and a substantial specific surface area, rendering it an optimal material for electrocatalysis. However, its inert basal planes account for a relatively large proportion, and the limited exposure of active edge sites restricts its further utilization and development. Here, we report a remote nitrogen (N)-plasma treatment that incorporates substituted N into monolayer MoS_2_ (ML-MoS_2_) and activates its basal plane for the hydrogen evolution reaction (HER). The optimized N-doped ML-MoS_2_ delivers an overpotential of 348 mV at 10 mA cm^−2^ and a Tafel slope of 94 mV·dec^−1^. Experimental and theoretical results show that nitrogen incorporation induces lattice defects and charge redistribution and increases active-site density, thereby enhancing HER activity. This plasma-based, controllable surface activation strategy provides a scalable route to tune the catalytic performance of two-dimensional transition-metal dichalcogenides.

## Highlights

• Remote N-plasma treatment enables controlled doping in monolayer MoS_2_
• N-doping activates the inert basal plane via charge redistribution
• Optimized MoS_2_ exhibits a low HER overpotential of 348 mV
• Calculations show reduced band gap and optimized ΔG_H_ of 0.10 eV

## Graphical abstract

**Figure:**
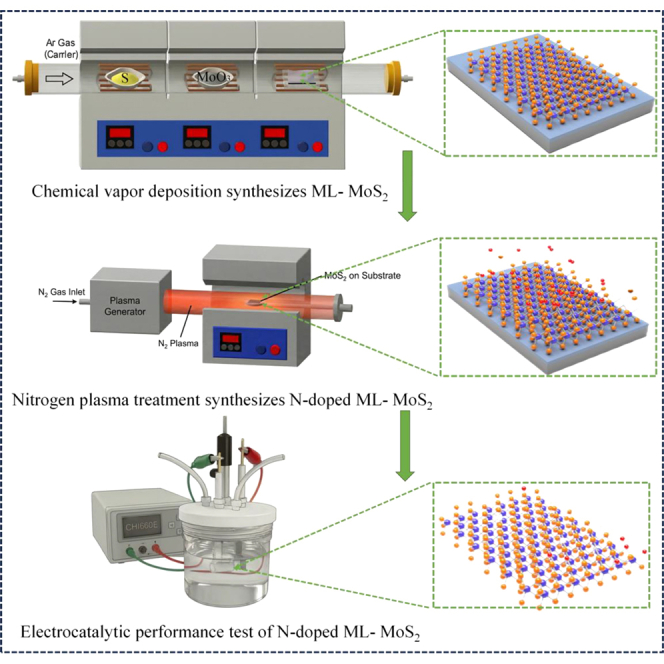

## Introduction

Hydrogen energy has emerged as an optimal alternative to mitigate global energy shortages and environmental degradation, due to its distinctive features. Its plentiful availability, elevated energy density, and cleanliness render it highly valued.^1^^,^^2^^,^^3^ The HER is essential for hydrogen synthesis, freeing us from dependence on carbon-based fossil fuels.^4^^,^^5^ Nonetheless, its elevated energy usage and diminished yield pose considerable concerns.^6^^,^^7^ Platinum (Pt) and Pt-based materials have been identified as the best electrocatalysts for HER thus far. However, while platinum-based noble metal catalysts exhibit excellent catalytic activity, their high cost and restricted availability limit their extensive industrial application.^8^^,^^9^^,^^10^ Thus, finding abundant, affordable, and effective non-noble metal catalysts is essential to hydrogen energy use. Recent findings show that molybdenum disulfide (MoS_2_) has catalytic activity equivalent to Pt, making it the most viable non-noble metal sulfide alternative to Pt.^11^^,^^12^^,^^13^ In particular, monolayer MoS_2_ with a layered structure exhibits unique surface, size, and macroscopic quantum tunneling effects, improving its HER efficiency.^14^^,^^15^ MoS_2_’s catalytic activity is thought to emanate from its edges, whereas its basal plane is very inert, limiting its practical applicability for HER.^16^^,^^17^^,^^18^ To address the MoS_2_ basal plane’s restricted catalytic activity, a variety of approaches have been devised, including phase engineering, interface electronic coupling, active unsaturated defects, and strain.^19^^,^^20^^,^^21^^,^^22^ Recent studies revealed that the incorporation of dopant atoms into the lattice of MoS_2_ by chemical doping techniques can enhance the electron density of both Mo and S atoms. This mechanism weakens Mo-S bonds and increases sulfur vacancies, improving the catalytic activity of the MoS_2_ basal plane.^23^^,^^24^^,^^25^ Currently, researchers are using nitrogen capable of stable integration into monolayer MoS_2_ to modify its electronic structure and enhance the electrocatalytic properties of HER. Nitrogen doping of MoS_2_ has been realized through methods including ammonothermal decomposition, chemical vapor deposition, and thermal annealing.^26^^,^^27^ These approaches, however, often involve complex procedures, limited environmental compatibility, and poor controllability over doping concentration and site specificity. To overcome these limitations, we present a facile and efficient plasma-treatment strategy that enables controlled incorporation of nitrogen atoms into the lattice of monolayer MoS_2_. As a result of electronic structure modulation and defect engineering induced by nitrogen doping, the inherently inert basal plane of MoS_2_ becomes activated for the HER.

## Results and discussion

### Structural characterizations

**Figure 1:**
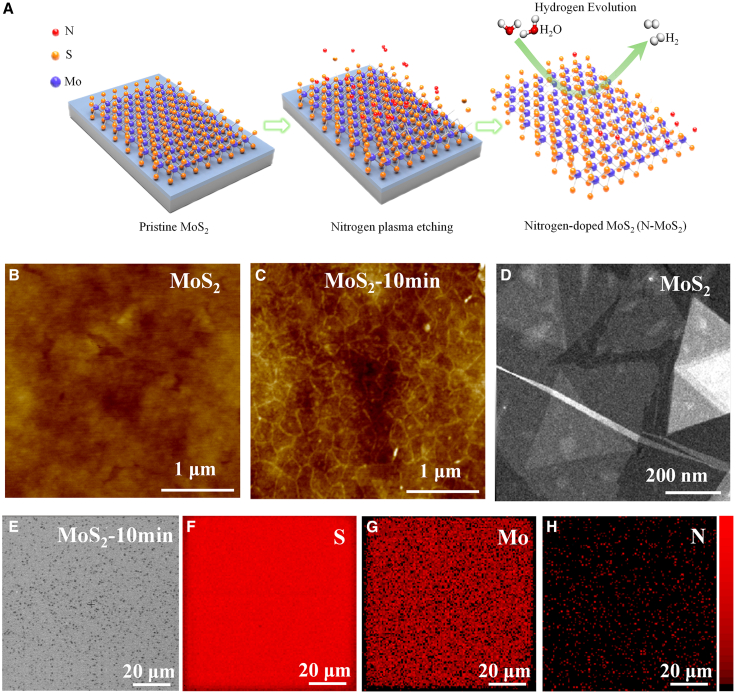
Preparation, morphological characterization, and elemental distribution of nitrogen-doped ML-MoS_2_ (A) Schematic diagram of nitrogen-doped ML-MoS_2_ preparation and its hydrogen evolution catalysis. (B) AFM characterization of pristine ML-MoS_2_. (C) Monolayer disulfide AFM characterization of nitrogen plasma treatment for 10 min. (D) Transmission electron microscopy (TEM) characterization of pristine ML-MoS_2_. (E) Scanning electron microscope image of nitrogen plasma treatment for 10 min of monolayer MoS_2_. (F–H) Elemental mapping images of S, Mo, and N in nitrogen plasma treatment for 10 min of monolayer disulfide.

Plasma doping is a surface treatment technique in which gases are heated to high temperatures and excited by an electric field or radiofrequency electromagnetic field to form plasma.^28^ This plasma comprises ionized gas molecules and free electrons, which possess high energy and reactivity. During the plasma doping process, high-energy ions are directed and accelerated through the plasma generator before being injected into the material surface. These injected ions penetrate the surface layer and integrate into the crystal structure, thereby altering the material’s chemical composition and crystal lattice.^29^^,^^30^^,^^31^^,^^32^^,^^33^ The schematic diagram for the preparation of nitrogen-doped MoS_2_ catalyst is shown in Figure 1A. The ML-MoS_2_ films were synthesized via chemical vapor deposition (CVD) and subsequently subjected to nitrogen plasma treatment. Further details are described in the Supporting Information. Under low-temperature and low-power conditions, extending the etching time of nitrogen plasma facilitates the incorporation of nitrogen atoms into the lattice of ML-MoS_2_. Atomic force microscopy (AFM) images of pristine ML-MoS_2_ and nitrogen-plasma-treated ML-MoS_2_ are shown in Figures 1B and 1C. Figure 1B clearly demonstrates that ML-MoS_2_ prepared by chemical vapor deposition exhibits high quality. As shown in Figure 1C, 10-min nitrogen plasma treatment leads to the generation of edge defects on the MoS_2_ surface. Correspondingly, the average surface roughness increased from 0.396 nm (pristine ML-MoS_2_) to 0.497 nm. The AFM images of MoS_2_ samples treated with nitrogen plasma for 5 min and 20 min are also shown in Figure S1. Figure 1D shows the TEM characterization of pristine ML-MoS_2._ Figures 1E–1H show the scanning electron microscope characterization and corresponding elemental mapping of MoS_2_ after low-temperature nitrogen plasma treatment for 10 min. The images demonstrate the uniform distribution of Mo, S, and N elements in ML-MoS_2_. Through Auger electron spectroscopy (AES) characterization, we found that the nitrogen doping content of the treated ML-MoS_2_ was 8.15% (Figure S2).

**Figure 2:**
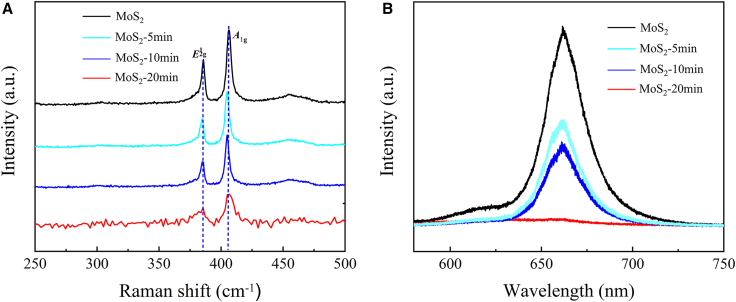
Raman and PL spectra of ML-MoS_2_ with the N-plasma treatment of different durations (A) ML-MoS_2_ Raman spectra of nitrogen plasma treatment at different times. (B) ML-MoS_2_ PL spectra of nitrogen plasma treatment at different times.

Raman spectroscopy (Figure 2A) provides clear evidence of progressive structural changes in monolayer MoS_2_ during nitrogen plasma treatment. The pristine MoS_2_ exhibited sharp E^1^_2_g (386.2 cm^−1^) and A_1_g (406.3 cm^−1^) peaks, indicative of a well-ordered hexagonal lattice. With increasing plasma exposure time from 5 to 20 min, both Raman modes progressively weaken and broaden, nearly vanishing at 20 min. These line-shape changes are indicative of increased lattice disorder under plasma exposure. Photoluminescence (PL) spectra (Figure 2B) further support this conclusion. Pristine MoS_2_ exhibited a strong excitonic PL peak, which decreased dramatically with longer plasma treatment due to the introduction of non-radiative recombination centers at defect sites. A concurrent blueshift was observed, suggesting that nitrogen incorporation modifies the electronic band structure, increasing the energy of radiative transitions.^34^^,^^35^^,^^36^^,^^37^ Plasma treatment first introduced defects (structural change), which exposed active sites and created favorable positions for nitrogen substitution.

**Figure 3:**
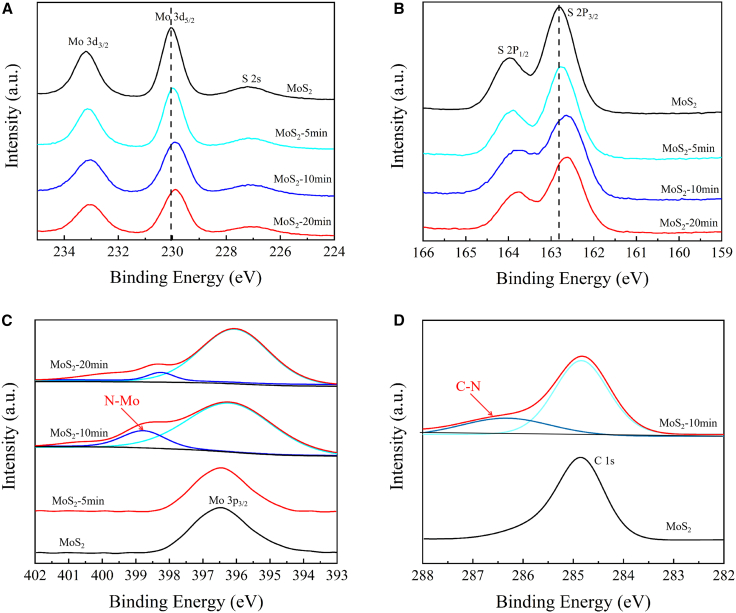
XPS profiles of pristine and nitrogen-plasma-treated monolayer MoS_2_ (A and B) XPS profiles of the Mo 3d (A) and S 2p orbitals for the ML-MoS_2_ and nitrogen plasma treated ML-MoS_2_ catalysts (B). (C) XPS profiles of the N1s orbitals for the ML-MoS_2_ and nitrogen plasma treated ML-MoS_2_ catalysts. (D) XPS profiles of the C 1s orbitals for the ML-MoS_2_ and nitrogen plasma treated ML-MoS_2_ catalysts.

Then X-ray photoelectron spectroscopy (XPS) analyses were used to reveal the chemical composition and stoichiometry of pristine and nitrogen-incorporated ML-MoS_2_ films, as depicted in Figure 3. Pristine ML-MoS_2_ exhibited two peaks in the Mo 3d spectrum at approximately 230 and 233.2 eV, corresponding to the doublet of Mo^4+^ 3d_5/2_ and Mo^4+^ 3d_3/2_ states.^38^ In the S 2P region, the doublet peaks of S 2p_3/2_ and S 2p_1/2_ appeared at 162.8 and 164 eV,^39^ respectively. The positions and shapes of Mo 3d and S 2p peaks confirm that ML-MoS_2_ exists solely in the semiconductor 2H phase. Following nitrogen plasma treatment, both Mo 3d and S 2p peaks exhibited a shift toward lower binding energies, as shown in Figures 3A and 3B. This is mainly attributable to charge transfer resulting from nitrogen doping and band bending mediated by the creation of Mo-N covalent bonds.^40^^,^^41^ The downward shift of these peaks also signifies that the Fermi level approaches the valence band maximum (VBM), which also aligns with the presence of nitrogen atoms in the treated ML-MoS_2_ films. Additionally, no chemical state of S-N bonds (164.8 eV) is detected in the S 2P region, indicating that nitrogen does not interact with sulfur. Figure 3C shows a low-intensity peak at 398.8 eV in the (N 1s) region, which is attributed to N-Mo bonds, confirming the presence of nitrogen atoms in ML-MoS_2_.^42^^,^^43^ Based on the correlation between the new chemical states in N 1s and Mo 3d, the formation of Mo-N bonds was determined. It is also important to note that a small amount of carbon adsorbed on the ML-MoS_2_ surface gives a low-intensity C 1s feature assignable to C-N interactions (Figure 3D). Subsequently, the S/Mo atomic ratios from the XPS spectra (charge-corrected to the C 1s peak at 284.8 eV, using standard sensitivity factors) were approximately 1.9:1 for pristine MoS_2_, 1.7:1 for MoS_2_-5 min, 1.6:1 for MoS_2_-10 min, and 1.7:1 for MoS_2_-20 min. Moreover, no detectable nitrogen incorporation occurred in the MoS_2_ monolayer during the initial 5 min of nitrogen plasma treatment. After 10 min of treatment, the nitrogen content reached approximately 9.35%, which is very similar to the 8.15% measured by AES reported in our previous study. However, when the plasma treatment time was further prolonged to 20 min, the nitrogen content decreased to 5.8%, indicating partial loss of incorporated nitrogen due to excessive plasma-induced etching and defect generation. The full spectral characterization of nitrogen-doped monolayer molybdenum disulfide is shown in the supporting information Figure S3. These findings revealed that by treating ML-MoS_2_ with nitrogen plasma, nitrogen atoms are incorporated into the ML-MoS_2_ lattice by substituting Sulfur atoms.^44^^,^^45^^,^^46^

## Hydrogen evolution reaction performance evaluation

**Figure 4:**
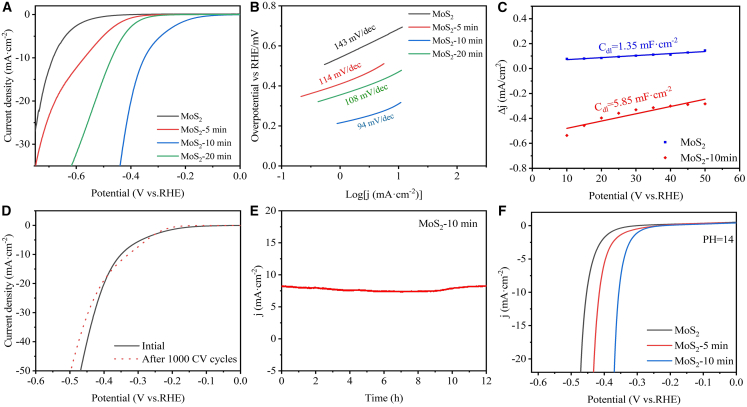
Electrocatalytic HER performance characterization of nitrogen-plasma-treated monolayer MoS_2_ (A) Linear scanning voltammetry characteristic curves of ML-MoS_2_ catalysts treated with ML-MoS_2_ and nitrogen plasma under acidic conditions. (B) Tafel curves for ML-MoS_2_ and nitrogen plasma treated MoS_2_ catalysts. (C) Double-layer capacitances (C_dl_) of MoS_2_ and MoS_2_-10 min (derived from the linear fitting of capacitive current vs. scan rate curves). (D) Stability measurements for 8.15% nitrogen-doped ML-MoS_2_ using accelerated degradation tests (1000 cycles, 50 mV s^−1^). (E) *I*-*t* curve of MoS_2_-10 min. (F) Linear scanning voltammetry characteristic curves of ML-MoS_2_, MoS_2_-5 min, and MoS_2_-10 min under alkaline conditions.

We thus investigated the effect of nitrogen incorporation on HER. ML-MoS_2_ electrodes for electrocatalytic HER testing were fabricated from pristine ML-MoS_2_ films and nitrogen-incorporated ML-MoS_2_ films.^47^^,^^48^^,^^49^ Polarization curves and Tafel plots of these samples were measured in 0.5 M sulfuric acid electrolyte using a standard three-electrode configuration (Figure S4). Figures 4A and 4B show the linear scanning voltammetry characteristic curves and the Tafel slopes of pristine ML-MoS_2_ and N-plasma-treated ML-MoS_2_ with treatment durations of 5 min, 10 min, and 20 min. The pristine MoS_2_ exhibits a high overpotential of 683 mV at 10 mA cm^−2^ and a Tafel slope of 143 mV·dec^−1^, indicating sluggish kinetics. The overpotential of MoS_2_ treated by nitrogen plasma for 5 min was significantly reduced to 567 mV at 10 mA cm^−2^, and the Tafel slope was 114 mV dec^−1^. Moderately N-doped MoS_2_ (10 min N-plasma treatment, 8.15% N) shows a dramatically reduced overpotential of 348 mV at 10 mA cm^−2^ and a Tafel slope of 94 mV·dec^−1^, reflecting significantly enhanced reaction kinetics. However, MoS_2_ treated for 20 min shows decreased activity (470 mV at 10 mA cm^−2^), demonstrating that excessive lattice damage disrupts the conductive network and active site distribution. Based on these results, we confirmed that nitrogen-doped ML-MoS_2_ contains more unsaturated coordination Mo atoms, allowing more H^+^ to be adsorbed and activating the nitrogen-doped ML-MoS_2_ catalyst.^50^^,^^51^^,^^52^^,^^53^ Additionally, the current density values were normalized to the geometric area of the monolayer MoS_2_ catalysts, which ensured complete and uniform coverage of the electrode surface (Supporting Information). To distinguish geometric contributions from intrinsic properties, we quantified the electrochemically active surface area (ECSA) via double-layer capacitance (C_dl_) extracted from capacitive current vs. scan-rate plots (Figure S5 in the Supporting Information). The N-doped sample (10 min) exhibited a C_dl_ value of approximately 5.85 mF cm^−2^, significantly higher than that of the pristine sample (∼1.35 mF cm^−2^), suggesting an increased ECSA following plasma activation (Figure 4C). As shown in Figure 4D, the electrochemical durability of nitrogen-doped MoS_2_ was evaluated by cyclic voltammetry (CV) versus the reversible hydrogen electrode (RHE) over 1000 continuous cycles at a scan rate of 50 mV s^−1^ within a potential window of 0 to −0.5 V (vs. RHE). The nearly overlapping polarization curves before and after cycling indicate negligible degradation, demonstrating the remarkable stability and durability of the nitrogen-doped MoS_2_ catalyst during prolonged HER operation. In addition, chronoamperometry conducted at a fixed potential exhibits a stable current density over approximately 12 h, thereby confirming the good stability of the N-doped MoS_2_ catalyst (Figure 4E). As shown in Figure 4F, the nitrogen-doped MoS_2_ also exhibits significantly enhanced HER catalytic performance under alkaline conditions (1 mol/L KOH solution). The overpotential of 5-min treated MoS_2_ is 415 mV, and that of 10-min treated MoS_2_ is 355 mV, both of which are notably lower than that of pristine MoS_2_ (453 mV). Under alkaline conditions, the N-doped sample (10 min) also exhibits a significantly higher C_dl_ than the pristine sample (Figure S6), consistent with an increased ECSA. Additionally, the N-doped sample also demonstrated excellent operational stability, maintaining a constant current density for ∼12 h under alkaline conditions. These results verify that nitrogen doping activates the basal-plane sites of MoS_2_, yielding efficient HER catalysis across a range of pH environments.

### Theoretical calculations

**Figure 5:**
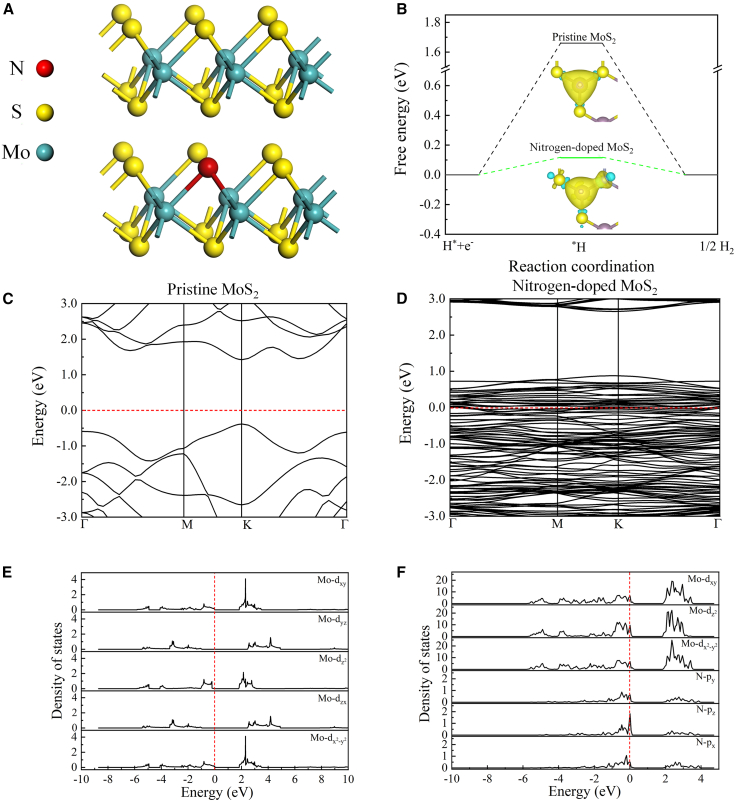
Structural models and electronic-structure calculations for N-doped monolayer MoS_2_ (A) Atomic models for ML-MoS_2_ and N-doped ML-MoS_2_ (8.30%). (B) The ΔG_H∗_ and CDD analysis of ML-MoS_2_ and N-doped ML-MoS_2_ (8.30%). (C) Electronic band structure of pristine 2H-MoS_2_ (1.79 eV, Fermi level indicated by a dashed line). (D) Electronic band structure of N-doped monolayer MoS_2_ (0.019eV, Fermi level indicated by a dashed line). (E) PDOS of pristine 2H-MoS_2_, showing Mo-d components. (F) PDOS of N-doped monolayer MoS_2_, showing Mo-d and N-p components.

To elucidate how nitrogen incorporation affects the HER activity of MoS_2_, we performed DFT calculations of the H adsorption free energy and electronic structure for pristine and N-doped monolayer MoS_2_. As shown in Figure 5A, we have established a molybdenum disulfide model and a molybdenum disulfide model with a nitrogen content of 8.30%. The Gibbs free energy of hydrogen adsorption (ΔG_H∗_) on Mo sites decreases significantly from 1.67 eV for pristine MoS_2_ to 0.10 eV following N doping (Figure 5B), suggesting a near-thermoneutral hydrogen adsorption/desorption energy, which is conducive to a lower hydrogen evolution reaction overpotential. Charge density difference (CDD) analysis, wherein yellow regions indicate electron accumulation, reveals substantial electron density enrichment around the adsorbed hydrogen (H∗) and a concomitant electronic redistribution across adjacent Mo, S, and N atoms. This electronic rearrangement signifies a tailored local Mo-H bonding environment that enhances the hydrogen adsorption state, as corroborated by electronic structure calculations that align with the observed energetic improvements. Figure 5C reveals that pristine 2H- MoS_2_ exhibits a semiconducting character, with the Fermi level located within a band gap of 1.79 eV. Nitrogen incorporation induces a substantial reduction in the band gap to a mere 0.019 eV, as demonstrated in Figure 5D. This drastic band gap narrowing signifies a significant increase in charge carrier density and is expected to markedly enhance interfacial charge transport properties. Projected density of states (PDOS) analyses further elucidate the orbital origins of the electronic modulation induced by N doping. In the pristine 2H-MoS_2_ system (Figure 5E), the five Mo-d sub-bands (d_xy_, d_yz_, d_xz_, d_x_^2^_-y_^2^, d_z_^2^) reside predominantly below the Fermi level, resulting in a low density of states (DOS) at the Fermi level. Upon N doping (Figure 5F), the d_xy_, d_yz_, and d_z_^2^ orbitals acquire substantial spectral weight near the Fermi level, accompanied by the emergence of N-p states in the same energy region. This indicates pronounced Mo-d-N-p hybridization, which enhances the metallicity of the system. The corresponding increase in DOS at the Fermi level, coupled with a concomitant weakening of the local Mo-S bonding, rationalizes the near-thermoneutralΔG_H∗_. These electronic structure modifications are consistent with the experimentally observed reduction in the HER onset overpotential and improved Tafel kinetics. Additionally, the orbital-resolved band centers of pristine and N-doped monolayer MoS_2_ were shown in Table S1 of the Supplementary Information. And the total density of states and site-projected PDOS plots for both systems, along with a comparative analysis of band centers, are provided in the Figure S7.

In summary, we successfully employed a plasma technique to introduce N atoms into the basal plane of ML-MoS_2_ and identified their role in HER electrocatalysis from experimental and theoretical views. N doping increases the electron density of Mo and S in MoS_2_ and weakens Mo-S bonds, which in turn leads to the formation of more sulfur vacancies on the ML-MoS_2_ surface. S vacancies in the basal plane not only can serve as additional active sites but also efficiently optimizeΔG_H∗_ As a result, N-doped ML-MoS_2_ (8.15% N) displays excellent catalytic properties (overpotential at 10 mA cm^−2^ of 348 mV, Tafel slope of 94 mV·dec^−1^). Our work presents a straightforward yet powerful strategy to activate the inert basal plane of MoS_2_, offering a guiding framework for the design and synthesis of other efficient two-dimensional transition-metal chalcogenide catalysts.

### Limitations of the study

While the remote nitrogen plasma treatment effectively activates the basal plane of MoS_2_ and enhances HER performance, several limitations should be noted. First, the process requires strict control over the exposure time, as excessive treatment leads to lattice damage and reduced nitrogen content, posing challenges for industrial scalability. Second, while we experimentally optimized the doping level, this study did not include a comprehensive theoretical analysis of how different doping concentrations affect the electronic structure andΔG_H∗_, but focused instead on the optimal configuration. Finally, the electrocatalytic performance was evaluated primarily in a three-electrode half-cell configuration. Although the catalyst demonstrates stability in acidic and alkaline media, its performance in a practical membrane-electrode-assembly electrolyzer under high current densities remains to be validated.

## Resource availability

### Lead contact

Further information and requests for resources should be directed to and will be fulfilled by the lead contact, Jianqi Zhu (jianqikitty@126.com).

### Materials availability

All stable materials generated in this study are available from the corresponding author upon reasonable request.

### Data and code availability

• All data reported in this article will be shared by the lead contact upon request.
• This article does not report original code.
• Any additional information required to reanalyze the data reported in this article is available from the lead contact upon request.

## Acknowledgments

This work is supported by the 10.13039/501100001809National Natural Science Foundation of China (12575311) and the 10.13039/501100018542Natural Science Foundation of Sichuan Province (2025ZNSFSC0867).

## Author contributions

Conceptualization, J.Z. and H.H.; methodology, H.H., A.W., and F.Y.; investigation, H.H., A.W., R.S., X.X., and Y.W.; formal analysis, D.Z., M.W., and Y.H.; writing original draft, J.Z., H.H., and A.W.; review and editing, J.Z., Z.X., and L.L.; visualization, H.H. and A.W.; supervision, J.Z.; funding acquisition, J.Z. and M.W.

## Declaration of interests

The authors declare no conflict of interest.

## STAR★Methods

### Key resources table

**Table:** 

REAGENT OR RESOURCE	SOURCE	IDENTIFIER
**Chemicals, peptides, and recombinant proteins**		

Sulfur powder	Alfa Aesar	7704-34-9
Molybdenum oxide (MoO_3_)	Alfa Aesar	MRM60
Sulfuric acid (H_2_SO_4_)	Chengdu Cologne Chemical	7664-93-9
Potassium hydroxide (KOH)	XIHUA	\

**Software and algorithms**		

Vienna ab initio simulation package	Kresse et al.^54^	https://www.vasp.at/
OriginLab	https://www.originlab.com/	Origin 2021

**Other**		

Ar gas (High purity)	Chengdu Longtai Industrial Gas	\
Nitrogen gas (High purity)	Chengdu Qiaoyuan Gas	\

### Experimental model and study participant details

This study does not involve human subjects or experimental models.

### Method details

The N-doped ML-MoS_2_ electrocatalysts were prepared using a sequential synthesis approach: (I) the initial growth of high-quality monolayer MoS_2_ films by CVD, followed by (II) the controlled incorporation of nitrogen atoms into the lattice using nitrogen plasma treatment. The overall synthetic route for the plasma-engineered MoS_2_ can be summarized as follows.

#### Synthesis of ML-MoS_2_

Here, we obtained single-layer MoS_2_ using chemical vapor deposition (CVD). A three zone furnace was used for CVD growth of MoS_2_. SiO_2_ (300 nm)/P++Si served as substrates. Sulfur(S) (Alfa Aesar 99.9%) and molybdenum trioxide (MoO_3_) (Alfa Aesar 99.999%) were used as precursors and loaded in zoneⅠand Ⅱ, respectively. The distance between the two sources was 22 cm. The substrates were put in the third zone. The temperatures of MoO_3_, S and substrates were 560, 120 and 780°C, respectively. Each temperature zone was kept stable for 20 min before growth. During the growth, argon was used as carrying gas at a flow rate of 130 sccm and the vacuum pressure was kept at 0.67 Torr.

#### Synthesis of N-doped ML-MoS_2_

The monolayer MoS_2_ films were treated with nitrogen plasma using a remote plasma system. The ignition power of the plasma generator was set to 50 W, the experimental temperature was 50 °C, and the gas flow rate was 70 sccm. Keeping these conditions constant, we adjusted the etching time of the nitrogen plasma to obtain nitrogen-doped monolayer MoS_2_ samples.

#### Characterization

The Raman spectra was collected on a Lab RAM HR Evolution (Horiba Jobin Yvon) micro-Raman system with an excitation laser wavelength of 532 nm (power of 0.2 mW). The surface characteristics and chemical states of the samples were investigated using a Thermo Scientific Escalab 250Xi X-ray photoelectron spectroscope (XPS). Binding energies were calibrated to the C 1s peak of adventitious carbon, which was set at 284.8 eV. The surface morphology and roughness were characterized by Atomic Force Microscope (AFM, Dimension Edge, BRUKER, U.S.A.) and Transmission Electron Microscope (TEM, Thermo Fisher-Tecnai G2 F30). The microstructural details and elemental distribution (Mo, S, and N) were obtained by Scanning Electron Microscope (SEM, Hitachi-Regulus 8230), equipped with Auger Electron Spectroscopy (AES, PHI 710).

#### Electrochemical measurements

The evaluation of HER performance was conducted using a CHI660E electrochemical workstation in a three-electrode system. The working electrode was a platinum foil (1×1 cm^2^), and the reference electrode was a saturated Hg/HgCl (Ag/AgCl) electrode. All measurements were performed in a N_2_-saturated 0.5 mol/L H_2_SO_4_ aqueous solution. The working electrode was prepared by transferring the monolayer MoS_2_ catalyst onto the surface of a glassy carbon electrode (GCE) with a geometric area of 0.15^2^ × π cm^2^ via a wet transfer technique, ensuring complete and uniform coverage of the electrode surface. And the geometric surface area is equivalent to the actual electrochemically active surface area. The electrochemical workstation was used to measure and study the electrocatalytic hydrogen evolution performance of the working electrode, with linear sweep voltammetry employed for scanning tests during the experiment.

All potentials in the electrochemical measurement are converted to potentials relative to the reversible hydrogen electrode (RHE) according to the following Equation 1:

(Equation 1) E (vs. RHE) =E (vs. Ag/AgCl)+0.0592×pH+0.1989V

The Tafel slope is obtained by fitting the polarization curve data with the Tafel Equation 2:

(Equation 2) η = a + b lg j

where a is the Tafel intercept, which is expressed as; the electrode overpotential value at zero current density; j is the current density; b is the required Tafel slope.

The electrochemically active surface area (ECSA) was calculated using Equation 3:

(Equation 3) ECSA=C_dl_/C_s_

where is the double-layer capacitance, and is the specific capacitance of a smooth-surfaced reference sample (glassy carbon electrode, GCE) under the same experimental conditions, with a value of 0.035 mF/cm^2^ (0.5 M H_2_SO_4_),0.04 mF/cm^2^ (1 M KOH).^55^

#### Computational methods

The spin-polarized density functional theory (DFT), implemented in Vienna ab initio simulation package (VASP),^2^ was employed for all computations in this work. To treat the interactions between electrons, the Perdew-Burke-Ernzerhof (PBE)^3^ within the generalized gradient approximation (GGA) was adopted. Meanwhile, the projector augmented wave (PAW)^4^ method was chosen to describe the electron–ion interactions. The van der Waals (vdW) interaction was taken into consideration with DFT-D3 method. The kinetic cutoff energy was set to 450 eV, and a vacuum space along z direction was fixed to 15 Å to avoid the interactions between periodic images. 5 × 5 × 1 and 21 × 21 × 21 Γ-centered Monkhorst-Pack k-point grids were applied for geometry optimizations. In addition, the convergence thresholds of energy and force were 10^-5^ eV/atom and 0.02 eV/Å, respectively.

The Gibbs free energies of the adsorbed hydrogen (H∗) on the basal planes of pristine ML-MoS_2_ and N-doped ML-MoS_2_ were calculated according to the following definition:

(Equation 4) ΔG_H∗_ = ΔE_H_ +ΔE_ZPE_-TΔS_H_

Wherein, ΔE_H_ is the chemical adsorption energy of the hydrogen atoms on the different MoS_2_ surfaces; ΔE_ZPE_ and ΔS_H_ represent the differences in zero-point energy and entropy, respectively, between the adsorbed H∗ and the gas phase H_2_.^56^

### Quantification and statistical analysis

We did not perform any statistical analysis to filter out relevant data.
