# Supplementary material for: Nitrogen plasma engineered MoS2 for catalyzing hydrogen evolution reaction
Source: iScience. 2026 Jan 16;29(2):114714. doi: 10.1016/j.isci.2026.114714 (PMC12874104; doi:10.1016/j.isci.2026.114714)
Supplement: Document S1. Figures S1–S7 and Table S1 [file mmc1.pdf]

iScience, Volume *29*

Supplemental information

Nitrogen plasma engineered MoS_2

for catalyzing hydrogen evolution reaction

Haoyang He, Ai Wang, Fengrui Yang, Rui Shu, Xinyan Xie, Yongheng Wen, Dong Zhao, Mao Wang, Yijia Huang, Zhengwei Xie, Ling Li, and Jianqi Zhu

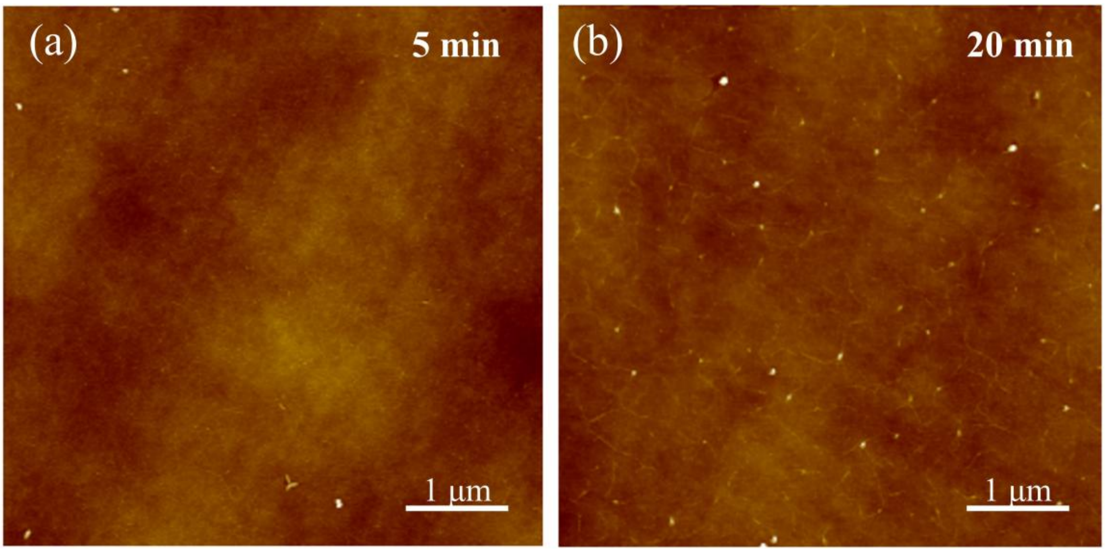

Figure S1. AFM images of MoS_2 after nitrogen plasma treatment. Related to Figure 1. (a) 5 min; (b) 20 min.

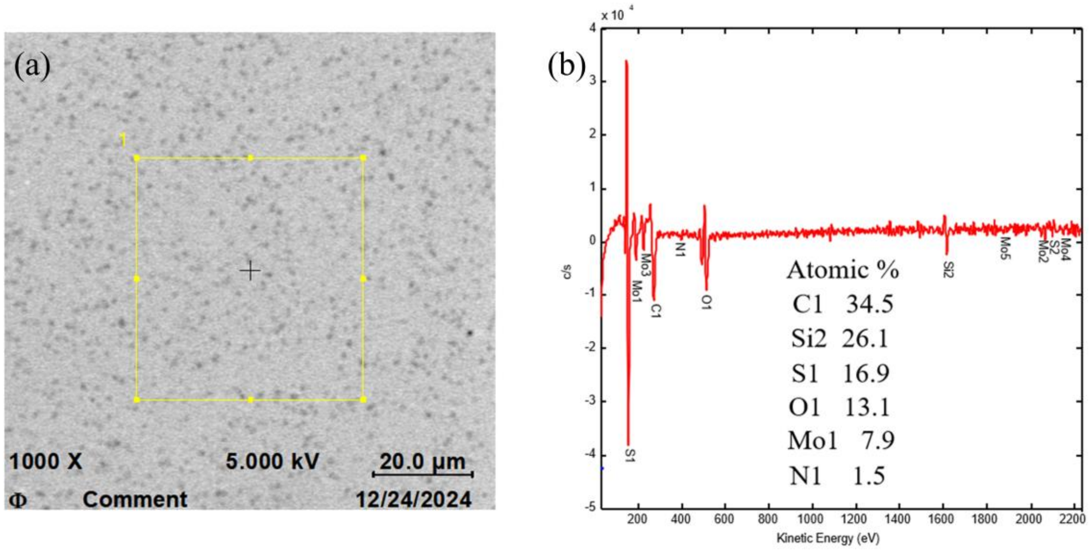

Figure S2. Morphology and AES analysis of nitrogen-doped MoS_2. Related to Figure 1. (a) SEM image of nitrogen-doped ML-MoS_2; (b) Differential AES spectra of nitrogen-doped ML-MoS_2.

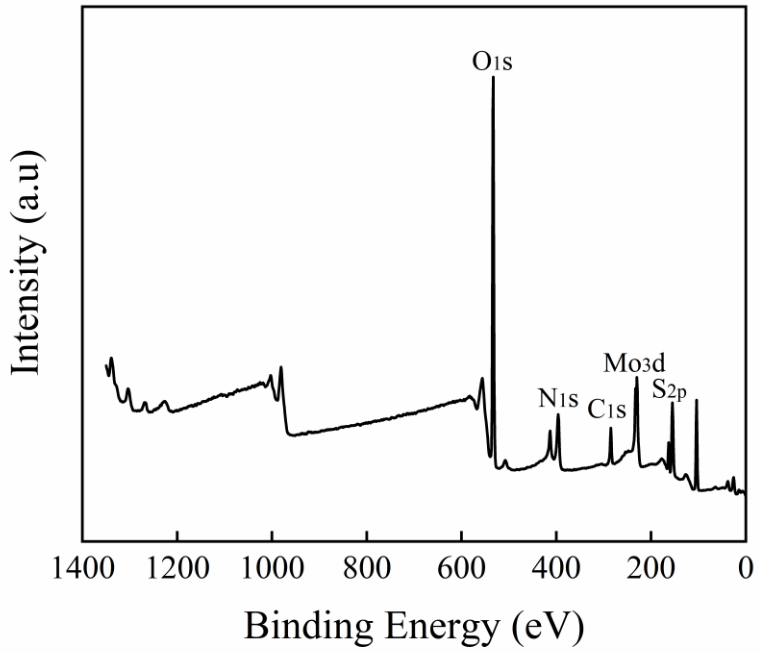

Figure S3. Full XPS spectra of nitrogen-doped ML-MoS_2. Related to Figure 3.

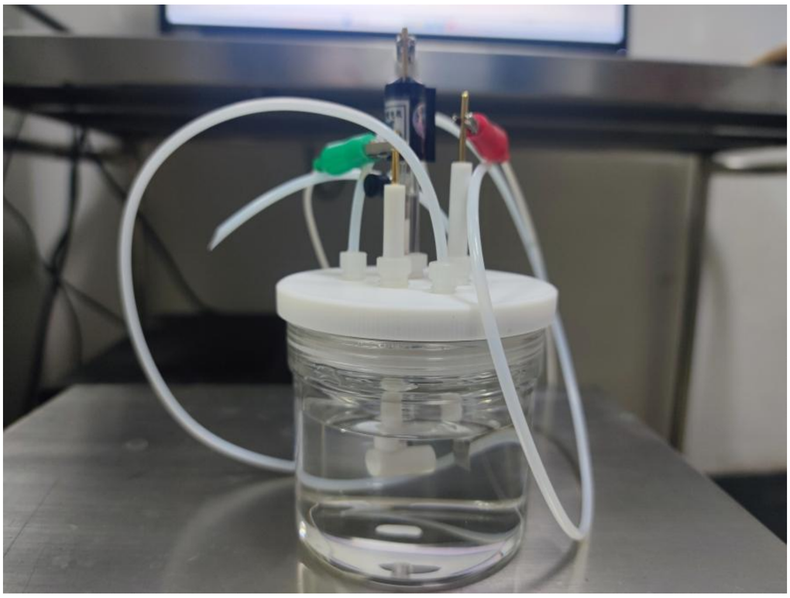

Figure S4. The standard three-electrodes configuration. Related to STAR Methods.

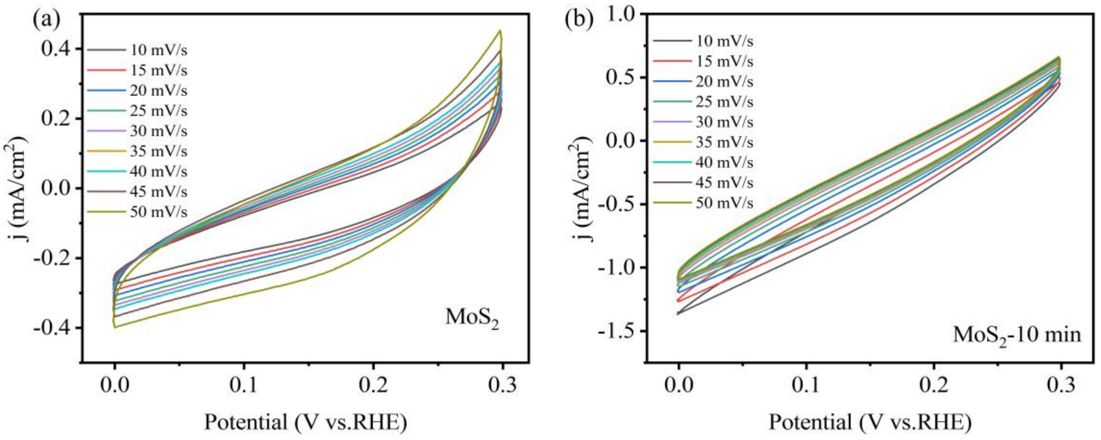

Figure S5. CV curves of pristine MoS_2 and MoS_2 treated with nitrogen plasma for 10 min at different scan rates under acidic conditions (pH = 0, 0.5 M H_2SO_4). The scan rates were set in the range of 10 mV/s to 50 mV/s, with 3 cycles conducted at each rate. The data from the third cycle were selected for analysis. Related to Figure 4. (a) MoS_2; (b) MoS_2 treated with nitrogen plasma for 10 min.

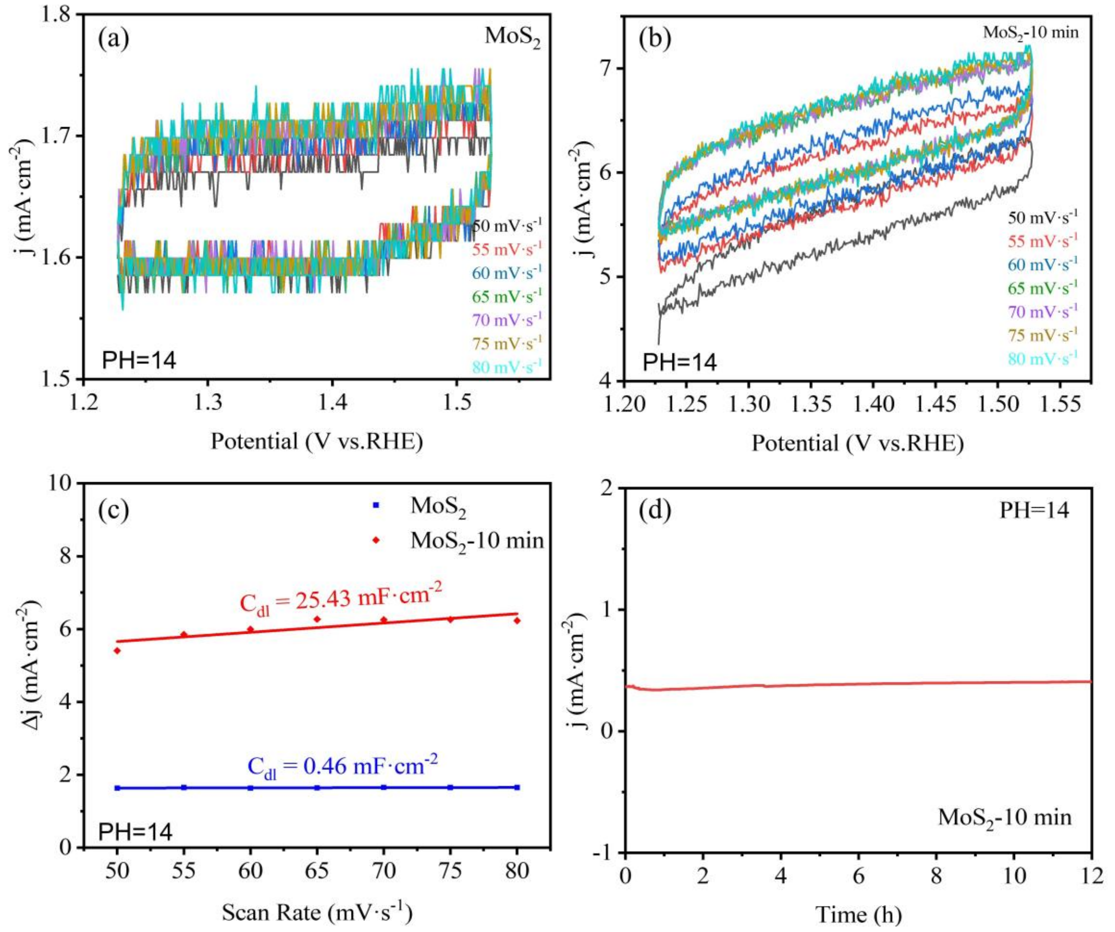

Figure S6. CV curves of MoS_2 and MoS_2 treated with nitrogen plasma for 10 min at different scan rates under alkaline conditions (pH = 14, 1 M KOH). The scan rates were set in the range of 50 mV/s to 80 mV/s, with 3 cycles conducted at each rate. The data from the third cycle were selected for analysis. Related to Figure 4. (a) MoS_2; (b) MoS_2 treated with nitrogen plasma for 10 min; (c) Double-layer capacitances (C_{dl}) of MoS_2 and MoS_2-10 min (derived from linear fitting of capacitive current vs. scan rate curves). Under alkaline conditions, the electrochemically active surface area (ECSA) of MoS_2 is approximately 11.5 cm^2, while that of the MoS_2-10 min is about 635.75 cm^2; (d) Chronoamperometric (I-t) curve of MoS_2 treated with nitrogen plasma for 10 min under alkaline conditions.

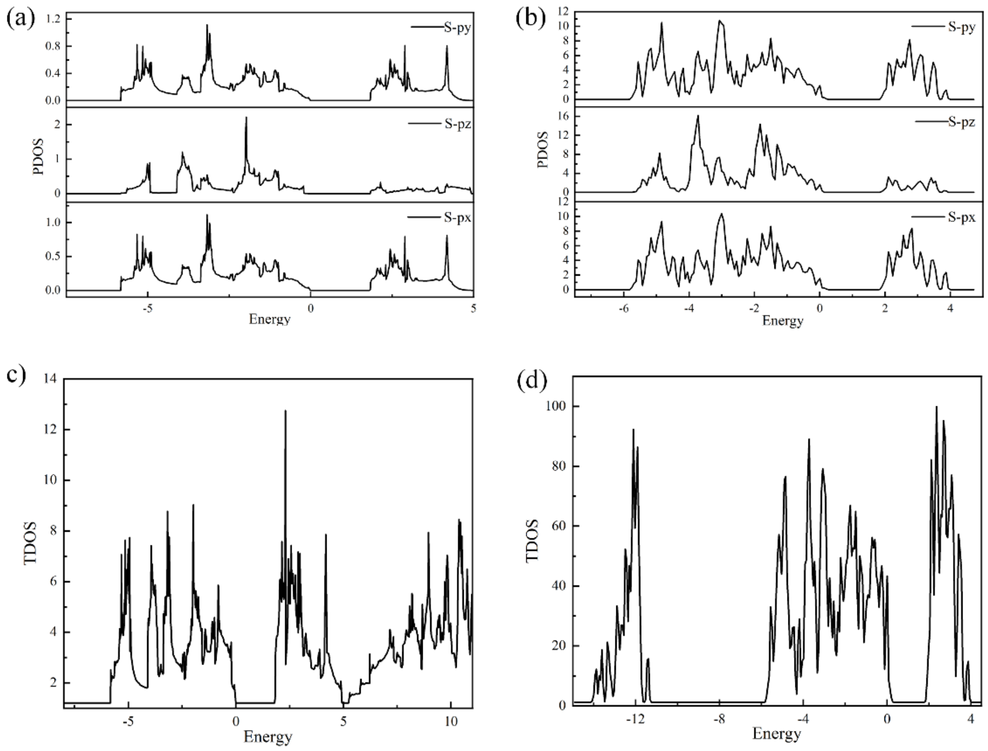

Figure S7. electronic-structure calculations for N-doped monolayer MoS_2. Related to Figure 5. (a) Projected density of states (PDOS) of pristine MoS_2, showing S-p components. (f) PDOS of N-doped monolayer MoS_2, showing S-p components. (c) Total density of states (TDOS) of MoS_2. (d) TDOS of N-doped monolayer MoS_2.

Table 1. Orbital-resolved band centers of pristine and N-doped monolayer MoS_2 (relative to E_F). Related to Figure 5.

System	Orbital	Band-center/eV
MoS_2	Mo-d	0.905
	S-p	-0.367
nitrogen-doped MoS_2	Mo-d	-0.414
	S-p	-1.821
	N-p	0.166
